# Advances in understanding and targeting eIF4E activity

**DOI:** 10.1042/BST20241045

**Published:** 2025-07-09

**Authors:** Sabrina D'Agostino, Caitlin Davies, Marissa V Powers, Paul A Clarke

**Affiliations:** Centre for Cancer Drug Discovery, The Institute of Cancer Research, London, U.K

**Keywords:** translation factors, cancer, therapeutics, translation, translation factors, cancer, drug discovery and design, molecular basis of health and disease

## Abstract

Eukaryotic translation initiation factor 4E (eIF4E) has long been recognised as a pivotal regulator of cap-dependent protein synthesis initiation. More recently, eIF4E has emerged as a multifunctional factor proposed to influence various aspects of RNA metabolism, including nuclear export of mRNA to the cytoplasm. Its versatile roles are largely attributed to its ability to bind the methyl-7-guanosine cap (m^7^G-cap) of mRNAs and participate in critical protein–protein interactions. Deregulated eIF4E expression or activity has been implicated in several diseases, but it is most prominently studied as an oncogene where its activity can drive cancer onset, progression and drug resistance. Consequently, eIF4E is a highly attractive target for the development of novel anti-tumour therapeutics. Recent advancements have provided new insights into the mechanism of action of eIF4E, leveraging fragment-based compound screening and genetically modified cell models to identify and characterise binding sites on this challenging-to-drug protein target. In this review, we summarise the multiple roles of eIF4E and features that underpin its activity in both the cytoplasm and nucleus, and the key findings related to the modulation of its activity and therapeutic potential.

## Introduction

Regulation of protein synthesis is critical, as it accounts for approximately a quarter of energy consumption in normal cells [1]. This process must be highly responsive, enabling cells to adapt swiftly to various physiological conditions and maintain cellular homeostasis through efficient protein production. Importantly, deregulation of protein synthesis is often associated with the onset and progression of various disorders, including cancer, neurodegenerative conditions and cardiovascular diseases [2–4]. Protein synthesis is a complex process involving multiple steps, requiring the co-ordinated activity of ribosomes, tRNAs, amino acids and regulatory proteins [5]. Among these steps, mRNA cap binding by eukaryotic translation initiation factor (eIF4E) and recruitment of initiation factors is a major regulatory checkpoint controlling translation efficiency.

Beyond its canonical role in translation initiation, eIF4E has also been implicated in post-transcriptional processes, including RNA capping, splicing and polyadenylation [6,7]. Additionally, eIF4E participates in the selective nuclear export of specific RNAs, leading Mars and colleagues to propose that eIF4E ‘terraforms’ the RNA landscape to support cellular needs [7]. Understanding the multifunctional role of eIF4E requires in-depth knowledge of its structure and its binding to different ligands. Structurally, eIF4E contains three highly conserved binding surfaces: 1. a ventral surface with the methyl-7-guanosine cap-binding pocket; 2. a dorsal surface involved in canonical protein–protein interactions (PPIs) with protein synthesis initiation factors such as eIF4G and 4E-BPs; and, 3. a lateral surface that mediates non-canonical PPIs. Recent findings suggest that effectively inhibiting eIF4E requires disruption of RNA cap binding or, alternatively, interfering with both canonical and non-canonical interactions with eIF4G [8].

The role of eIF4E in human disease is significant, particularly in cancer, where it acts as an oncogene. Overexpression of eIF4E has been demonstrated in tumours, and it has oncogenic activity in *in vitro* and *in vivo* models of cell transformation, which has made eIF4E a prominent therapeutic target [8,9]. While significant progress has been made in understanding eIF4E’s cellular role, questions remain regarding the balance between its roles in mRNA export and translation and its function within the translation initiation complex. The development of potent, highly selective tool compounds that directly modulate eIF4E’s different cellular functions would complement biophysical, biochemical and genetic approaches to study its role in both normal and disease cell models. For example, screening efforts have identified small molecules that block the eIF4E–eIF4G interaction [9]. However, these compounds currently exhibit poor drug-like properties, and so far, none have proven to be effective chemical tools or advanced to clinical trials (reviewed in [10,11]).

This review explores the multifunctional nature of eIF4E, with a focus on its structural interactions and regulatory roles. We summarise current research efforts aimed at refining our understanding of the molecular mechanisms of eIF4E activities, which could inform the development of novel and more effective therapeutic strategies for cancer and other human diseases.

### eIF4E and RNA processing

#### eIF4E and m^7^G capping

**Figure 1:**
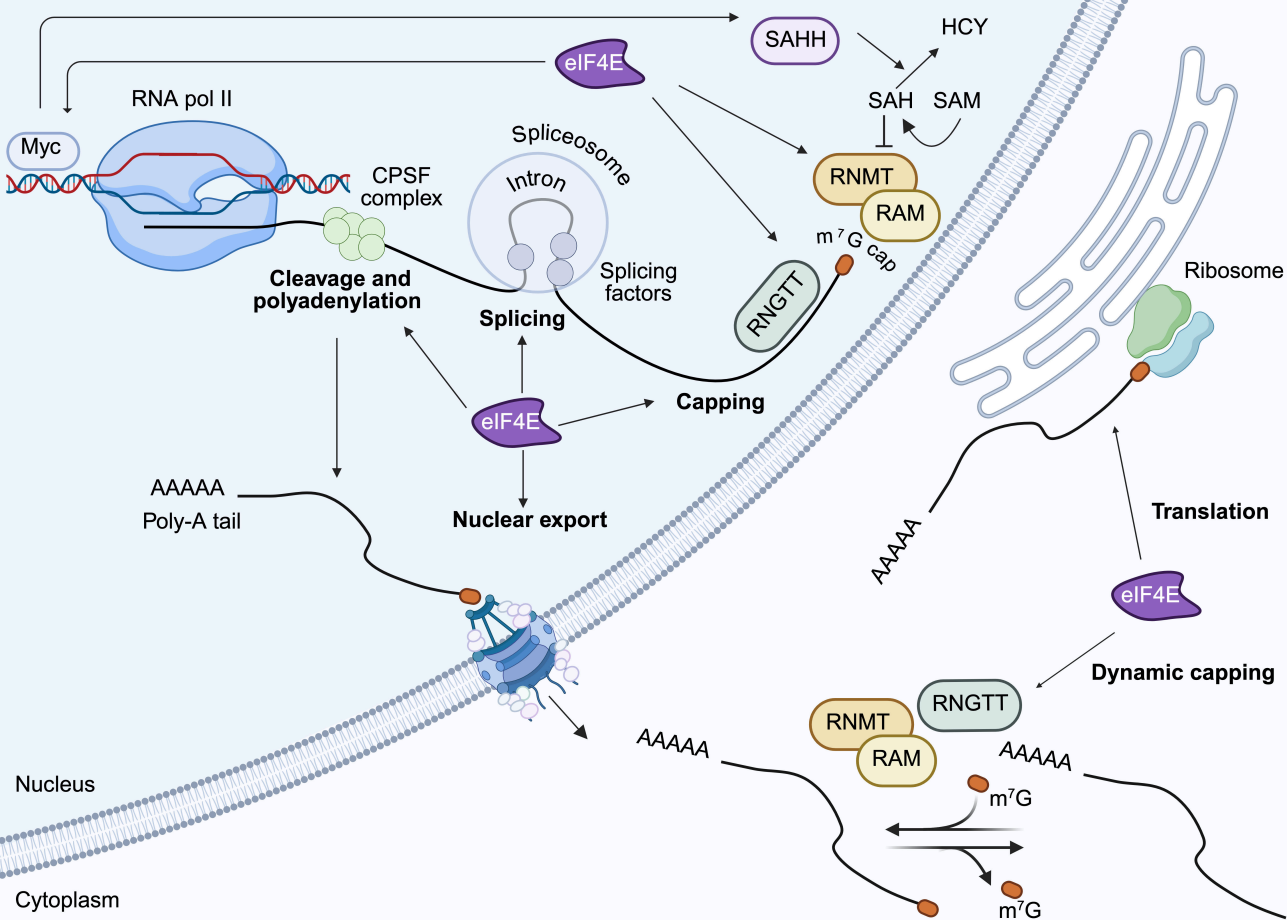
Potential role of eIF4E in different stages of RNA maturation. In eukaryotic cells, mRNA synthesised in the nucleus undergoes extensive modifications to produce a mature transcript capable of nuclear transport and subsequent translation in the cytoplasm. These RNA processing steps include the addition of the 5' m^7^G cap – a dynamic modification dependent on RNGTT (RNA guanylyltransferase and 5’ phosphatase), RNMT (RNA guanine-7-methyltransferase) and its cofactor RAM (RNMT activating mini-protein) using S-adenosyl methionine (SAM) as the methyl donor that can also occur in the cytoplasm to regulate translation – splicing, RNA cleavage within the 3' untranslated region, 3' polyadenylation, nuclear transport (see Figure 2 for details) and translation into protein (see Figure 3 for details). eIF4E plays a key role in regulating gene expression by co-ordinating RNA processing and translation to adapt to cellular demands (see text for more details), for example, eIF4E increases capping and translation of c-MYC transcripts which includes S-adenosylhomocysteine hydrolase (SAHH) which catalyses the hydrolysis of S-adenosylhomocysteine (SAH), an inhibitory by-product of RNMT methyltransferase activity, to L-homocysteine (HCY). Created in BioRender. https://BioRender.com/p75q573.

The m^7^G cap is a crucial 5'-epi-transcriptomic modification in eukaryotic mRNA that provides stability and protection against degradation [12,13] and serves as a binding site for proteins, such as eIF4E, facilitating mRNA nuclear export and translation (Figure 1). Capping is an active process dependent on RNGTT (RNA guanylyltransferase and 5' phosphatase) and RNMT (RNA guanine-7-methyltransferase), both of which are essential for eukaryotic cell survival (reviewed elsewhere [14,15]). Recent methodological advances have revealed that capping levels for certain RNAs are often below the expected 100%, challenging the notion of capping as a static housekeeping modification [14,16]. Instead, RNAs can undergo dynamic cycles of capping and de-capping, regulated by cell differentiation stage, development or oncogene expression [14,17–19].

Overexpression of eIF4E has been shown to enhance the capping efficiency of select transcripts from less than 40% to nearly 90% suggesting that eIF4E has roles beyond merely protecting the m^7^G cap [16]. Specifically, eIF4E overexpression increases the levels of RNGTT, RNMT and its co-factor RAM (RNMT activating mini-protein) by promoting nuclear export and the translational efficiency of their mRNAs [16]. RNMT directly interacts with the dorsal surface of eIF4E, a region also known to bind factors critical for translation and nuclear export, including eIF4G and 4E-BP1 [20]. Interestingly, the binding sites of eIF4E and RAM on RNMT overlap, suggesting potential competition between these protein interactions. However, the broader implications of the dynamics of eIF4E/RNMT binding remain to be fully elucidated.

In cancer cells, eIF4E boosts both the capping and translation of oncogenic transcripts including c-MYC, MDM2, CTNNB1 and CCND1 [16,21]. Downstream of eIF4E, elevated c-MYC stimulates RNMT activity by increasing expression of S-adenosylhomocysteine hydrolase (SAHH), that counters the inhibitory effects of S-adenosylhomocysteine (SAH), a by-product of RNMT methyltransferase activity (Figure 1) [22,23]. These data suggest that eIF4E-dependent capping may be an important node linking transcription and translation that could be exploited to inhibit cancer through pharmacological inhibition of eIF4E

#### eIF4E and splicing

Following 5' capping, many pre-mRNAs are processed by the spliceosome, which removes introns and ligates adjacent exons to produce mature mRNA [24,25] (Figure 1). Further diversification of the transcriptome and proteome can occur through alternative splicing. This is a tightly regulated process that varies the inclusion of exons in the final mRNA transcript and enables organisms to adapt to developmental and environmental cues by generating multiple protein isoforms from a single gene [24–27].

A recent study showed that eIF4E overexpression significantly alters splice site selection in 100–1000s of pre-mRNAs, particularly in cancer cells [28]. These data suggest a model where eIF4E exerts indirect effects on splice site selection by enhancing the nuclear export and translation of mRNAs encoding splicing factors while also influencing splicing directly through its interactions with specific pre-mRNAs or components of the spliceosome [28]. Further investigation will be needed to elucidate the precise mechanistic role of eIF4E in splicing regulation and to explore whether these effects extend beyond cancer contexts.

#### eIF4E mRNA cleavage and polyadenylation

Cleavage and polyadenylation (CPA) are essential steps in the maturation of RNA transcripts. CPA involves cleavage of the pre-mRNA within the 3' untranslated region (UTR) downstream of a polyadenylation signal (PAS) by the endonuclease CPSF3. Subsequent polyadenylation by poly(A) polymerase enhances transcript stability, facilitates nuclear export and promotes translation (Figure 1) [29,30]. Alternative polyadenylation (APA) adds another layer of diversity in the transcriptome and proteome by utilising different PAS sites within the 3' UTR, introns or exons. APA generates transcript isoforms with varying coding sequences or 3' UTR lengths, affecting transcript stability, localisation and translation efficiency and potentially leading to proteins with altered functions [29,30].

Emerging evidence suggests that eIF4E may play a regulatory role in CPA and APA, particularly in cancer [29–32]. Expression of eIF4E can promote the generation of mature transcripts that are more efficiently exported and translated [29–33]. Similar to its impact on splicing, eIF4E’s role in CPA and APA could be indirectly mediated by altered nuclear export or translation of mRNAs encoding CPA components or via interactions with the CPA machinery to enhance 3'-end CPA of specific RNAs [32]. Further research will be necessary to clarify the involvement of eIF4E and to determine broader implications in cancer or normal cell contexts.

### eIF4E-dependent nuclear export of mRNAs and nuclear trafficking of eIF4E

RNA export is a highly regulated process [34–38], requiring m^7^G cap addition, mRNA splicing and 3' polyadenylation [39–41]. This process controls the cytoplasmic availability of transcripts for protein translation and serves as a critical mechanism for mRNA surveillance [7,42]. The selective export of specific mRNA subsets, determined by conserved cis-acting elements called Untranslated Sequence Elements for Regulation (USER) codes, enables dynamic changes in the cellular proteome in response to cellular stimuli [43–45]. Perhaps not surprisingly, deregulation of mRNA export has been implicated in human disease [7,36,46–48].

**Figure 2:**
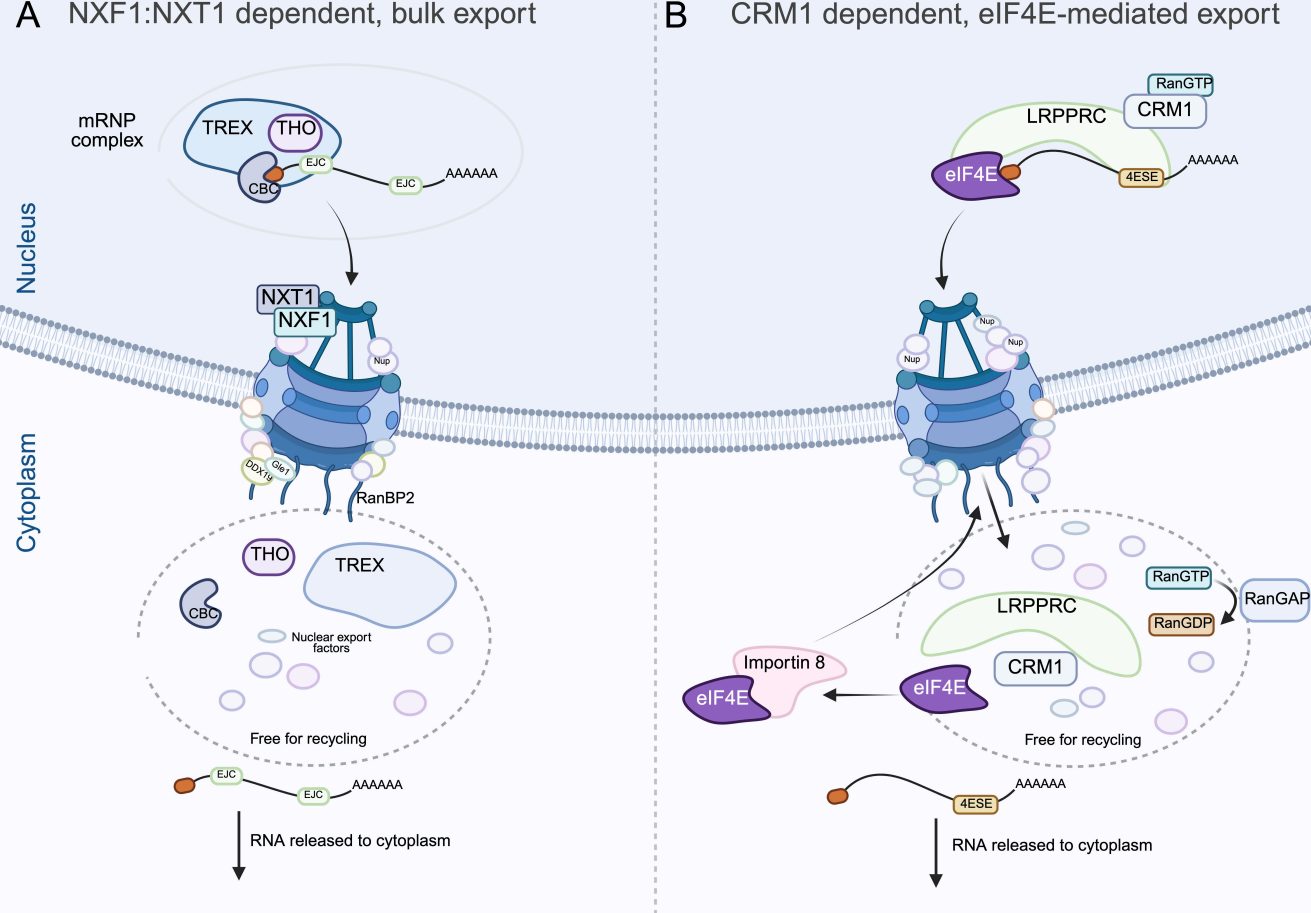
Nuclear export of RNA. **A**) The transcription export (TREX) complex (comprising UAP56, CIP29 and Aly/Ref proteins) is recruited to and assembled around the 5' cap-binding complex (CBC) of mRNAs containing exon-junction complexes (EJCs). Together with the THO multi-subunit complex, TREX bridges interactions between the target mRNA and the NXF1:NXT1 receptor complex facilitating transport through the nuclear pore complex (NPC). Upon reaching the cytoplasmic face of the NPC, cargo mRNA release is mediated by the ATP-dependent DEAD-box helicase DDX19 and its co-factor Gle1, allowing mRNA transcripts to diffuse into the cytoplasm for translation by ribosomes. Subsequently, the major cytoplasmic fibril component RanBP2 plays a key role in recycling export-factors back into the nucleus. **B**) RNAs containing 4ESE USER codes follow an alternative export pathway driven by eIF4E and the Chromosomal Maintenance 1/Exportin 1 (CRM1/XPO1) export receptor in a Ran-dependent manner. CRM1 does not bind mRNA directly but instead recognizes leucine-rich nuclear export signals within shuttling and adaptor proteins, which in turn associate with RNA or RNA-binding proteins. In eIF4E-specific export, the RNA-binding protein human antigen leucine-rich pentatricopeptide repeat containing protein (LRPPRC) binds both the 4ESE and cap-bound eIF4E. To facilitate CRM1-mRNP complex export, CRM1 directly interacts with nucleoporins (Nups) at the nuclear basket of the NPC. In the cytoplasm, Ran GTPase-activating protein (RanGAP) promotes GTP hydrolysis, leading to the dissociation of CRM1 from its cargo and subsequent mRNA release. Finally, cap-free eIF4E is targeted by Importin 8 for re-import into the nucleus [7,36]. Created in BioRender. https://BioRender.com/j26w708.

During export, co-transcriptional recruitment of export factors results in the formation of large RNA-associated messenger ribonucleoprotein (mRNP) complexes that accumulate in the interchromatin compartment [49–52]. Most RNAs are substrates of the NXF1:NXT1 mediated bulk export pathway [53,54], while a subset of RNAs utilise an alternative export pathway involving the CRM1/XPO1 complex (Figure 2) [54–56].

eIF4E contributes to gene expression regulation by directly mediating mRNA export [7,57–60]. Mutagenesis studies have highlighted the distinct role of eIF4E in nuclear export. For instance, the S53A mutation retains translation initiation activity but disrupts mRNA export and the oncogenic transformation activity of eIF4E [61–63]. However, not all residues and interactions are essential for mRNA export. For example, the W73A mutant that disrupts eIF4G binding retains export activity, suggesting that export and translation initiation mechanisms can be separated [57,60].

Approximately 3,500 mRNAs have been identified as potential eIF4E nuclear export targets. These mRNAs contain a ~ 50-nucleotide eIF4E sensitivity element (4ESE) USER code in their 3' UTRs [64]. This 4ESE, together with the 5' m^7^G cap, is sufficient to drive eIF4E-dependent mRNA export [58,64]. The eIF4E-dependent export pathway relies on the leucine-rich pentatricopeptide repeat containing protein (LRPPRC) within the CRM1/XPO1 complex. LRPPRC binds directly to the 4ESE (Figure 2) [60,64,65] and recruits eIF4E, which interacts with the m^7^G cap of target mRNAs [7,58,64]. LRPPRC associates with the dorsal surface of eIF4E through its N-terminus, while simultaneously engaging the CRM1 receptor, allowing the export of the LRPPRC-4ESE-eIF4E complex [64]. Knockdown of LRPPRC reduces eIF4E’s ability to bind 4ESE-containing mRNAs and inhibits eIF4E-dependent export, highlighting its key role in this pathway [60]. Certain co-factors are shared between the NXF1 and eIF4E/CRM1/XPO1 pathways, suggesting cross-talk between these pathways [60]. Meanwhile, endogenous 4ESE-containing mRNAs with long 3' UTRs containing competing USER codes can use both the bulk and eIF4E-dependent pathways [57–59].

Once in the cytoplasm, eIF4E is recycled back to the nucleus by Importin 8, which binds the positively charged region of the cap-binding site [7,66]. Mutation of the positively charged R157, K159 and K162 residues in the cap-binding site significantly reduces the affinity of eIF4E for Importin 8. This contrasts with the mutation of W56, which disrupts mRNA cap-binding and RNA export [58], but has no impact on importin 8 binding, indicating that the positive charge, rather than the entire cap-binding site, is critical for Importin 8-mediated nuclear import [66]. The mutually exclusive binding of Importin 8 and the m^7^G cap ensures that eIF4E export complexes release their mRNA cargo before eIF4E is recycled to the nucleus. Importantly, eIF4E actively involved in translation cannot be imported back into the nucleus [66]. Loss of Importin 8 leads to cytoplasmic accumulation of eIF4E, nuclear retention of 4ESE-containing mRNAs, reduced protein levels encoded by these mRNAs and impaired eIF4E-mediated oncogenic transformation [66]. Interestingly, RNA-free LRPPRC is also recycled via Importin 8, and eIF4E promotes the mRNA export of Importin 8 itself, regulating its cytoplasmic levels and mRNA targets [64].

### Cap-dependent translation

**Figure 3:**
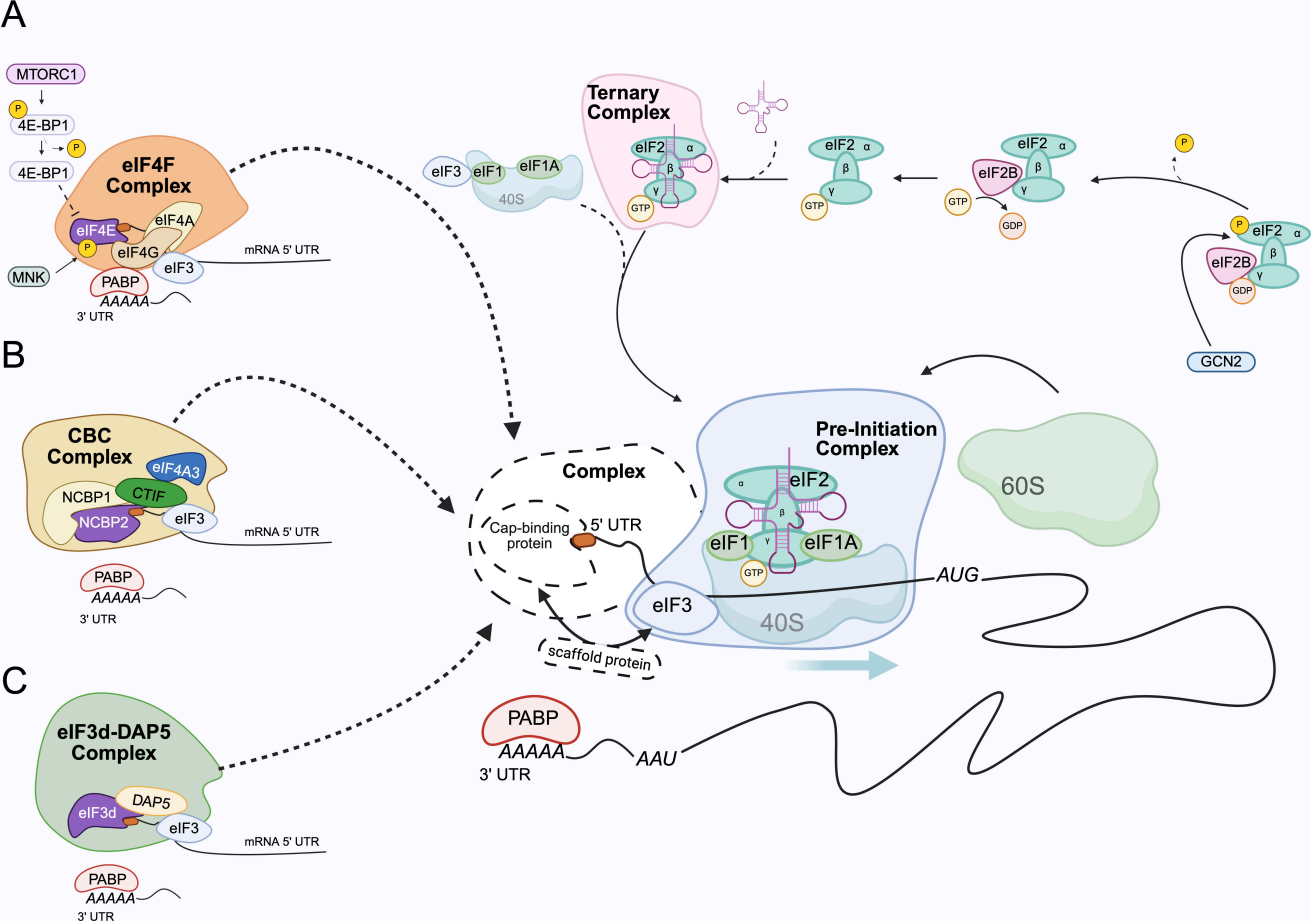
Cap-dependent protein translation. Protein synthesis is a complex process involving the co-ordinated activity of ribosomes, tRNAs, amino acids and regulatory proteins [5]. It can be divided into several steps: 1) mRNA cap binding and recruitment of initiation factors, 2) assembly of the ribosome at the mRNA initiator codon, 3) polypeptide chain elongation by the ribosome, 4) termination of elongation and ribosome release from the mRNA and 5) ribosome recycling for subsequent rounds of translation. Among these steps, initiation is the primary regulatory checkpoint controlling translation efficiency. **A**) Translation initiation begins with the binding of eIF4E to the m^7^G cap structure of the mRNA to induce eIF4F complex formation (eIF4E-eIF4G-EIF4A). Once bound to the 5' mRNA, eIF4E recruits eIF4G to its dorsal surface. eIF4G acts as scaffold protein for recruitment of eIF4A RNA helicase which unwinds RNA secondary structure and promotes the binding of the 43 S pre-initiation complex (PIC) through eIF3. The poly(A)-binding protein (PABP) also binds to the eIF4F complex via eIF4G forming a circular mRNA and enhancing translation. 4E-BP1 competes with eIF4G for binding to the dorsal surface. When hypo-phosphorylated, 4E-BP1 shows a higher affinity for eIF4E impairing the interaction with eIF4G, hence disrupting the formation of eIF4F complex. mTOR protein kinase indirectly regulates eIF4E function by phosphorylating 4E-BP1. When phosphorylated, 4E-BP1 is released from eIF4E which can, in this way, interact with eIF4G for the eIF4F complex formation. MNK protein kinase can directly phosphorylate eIF4E on S209 increasing eIF4E activity. **B**) The cap-binding complex (CBC) can mediate eIF4E-independent, cap-dependent translation of specific mRNAs. The CBC comprises two key components: nuclear cap-binding protein 2 (NCBP2), which directly binds to the 5'-cap structure, and nuclear cap-binding protein 1 (NCBP1) which associates with NCBP2. CBC binding to RNA occurs in the nucleus, this complex is exported to the cytoplasm following splicing and poly-adenylation of the CBC-associated mRNAs. CBC-bound mRNAs interact with CTIF (CBC-dependent translation initiation factor), which facilitates recruitment of the 40S ribosomal subunit via association with the eIF3 complex. In contrast with eIF4G, CTIF cannot bind PABP. To date, there is no known poly(A)-binding protein specific for the CBC cap-dependent translation. **C**) DAP5-eIF3d complex is a previously unknown cap-dependent translation mechanism needing further investigation to better understand which proteins are involved in the formation of this complex. To date it is known that subunit eIF3d, from complex eIF3, can directly bind the 5'-cap of several mRNAs recruiting DAP5 protein, also known as eIF4G2, which is an eIF4GI homologous that can bind eIF4A but not eIF4E and PABP because it is lacking the N-terminal domain.

The cytoplasmic function of eIF4E primarily revolves around regulating cap-dependent translation initiation (Figure 3A). Upon binding to the 5' cap of mRNAs, eIF4E initiates the formation of the eIF4F complex by recruiting eIF4G [7]. eIF4G, a scaffold protein, facilitates the recruitment of the helicase eIF4A, which unwinds secondary mRNA structures, enabling ribosome binding to the 5' UTRs [67]. The interaction between eIF4E and eIF4G can be inhibited by the repressor protein 4E-BP1 (4E binding protein), which competes with eIF4G for the same binding site on eIF4E [68–70]. When eIF4E binds to unphosphorylated 4E-BP1, the assembly of the eIF4F complex is inhibited [71].

4E-BP1 phosphorylation is regulated by the mechanistic target of rapamycin (mTOR), a protein kinase that exists in two functionally distinct complexes (mTORC1 or 2). mTORC1 is directly responsible for regulating 4E-BP1 activity and is activated in response to growth factors, nutrients or favourable energy conditions. Depending on the activating stimulus, mTORC1 phosphorylates 4E-BP1 at multiple different serine and threonine residues (Thr37, Thr46, Ser65 and Thr70). This phosphorylation reduces the affinity of 4EBP1 for eIF4E, causing it to dissociate from eIF4E, freeing eIF4E to interact with eIF4G and form the eIF4F complex. Inhibition of mTORC1 following stress, nutrient deprivation or treatment with pharmacological inhibitors prevents 4EBP1 phosphorylation, enabling 4E-BP1 to tightly bind and sequester eIF4E, preventing binding to eIF4G and effectively repressing eIF4E-dependent initiation. Through this mechanism, mTOR acts as a molecular switch to integrate upstream signals and finely tune protein synthesis [72].

**Figure 4:**
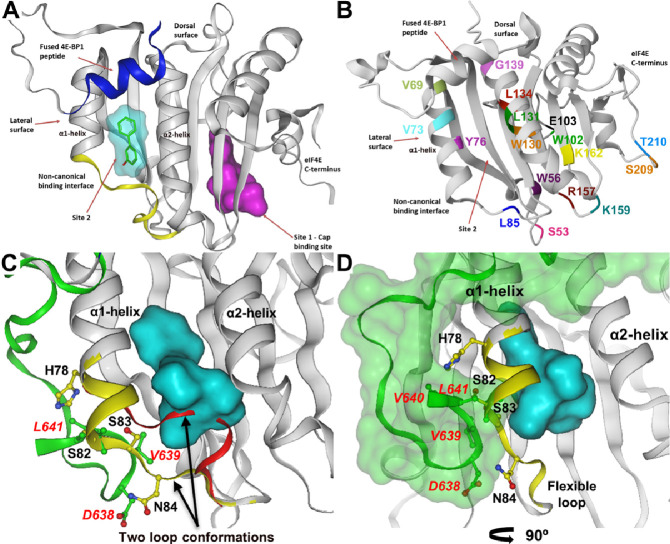
eIF4E X-ray structure and associated mutations. **A** ) The secondary structure of eIF4E is shown in ribbon representation (PDP: 8QM4). The majority of the protein (grey), canonical peptide derived from 4E-BP1 attached to the N-terminus of eIF4E (blue), loop region between the C-terminus of the α1-helix and N-terminus of the β3-sheet (yellow). The fragment hit bound in site 2 is depicted as green lines. Key features are highlighted, including the location of site 2 in comparison with the cap-binding site (the Cap-site ligand (m7-GTP) is shown for illustration purposes only. The surface representations of the fragment and m7-GTP are shown in cyan and magenta, respectively **B** ) X-ray crystal structure of eIF4E protein (PDB: 8QM9) overlaid with eIF4G peptide as shown. The location of residues selected for mutational analysis are highlighted. **C** ) Overlay of the eIF4G peptide (green ribbon) with the protein conformation of compound **4** bound eIF4E (grey ribbon) showing the two flexible loop conformations for compound **4** bound and unbound eIF4E (PDB: 8QM9). The reorganisation of α1-helix and the flexible loop region following compound **4** binding is displayed as a yellow ribbon, the Connolly surface of compound **4** is displayed in cyan. the original flexible loop conformation from the PDB: 5T46 structure of eIF4E bound to an eIF4G peptide is displayed as a red ribbon. **D** ) Overlay of eIF4G peptide (green ribbon and green Connolly surface) with the protein conformation of compound **4** bound eIF4E. The orientation has been rotated by~90° in the horizontal plane compared with that shown in the bottom left panel. figure adapted from [8].

The interaction of eIF4E with the m^7^G cap relies on cation-π interactions involving two conserved tryptophan residues (W56 and W102) and a glutamate residue (E103) [10,68]. Key charged residues (R157, K159 and K162) in the cap-binding pocket also help stabilise interactions with the m^7^G cap through hydrogen bonding and electrostatic interactions (reviewed in [7]). Substituting W56, W102 or E103 with leucine (L) or alanine (A) abolishes the cap-binding activity of eIF4E, while the W56A mutant disrupts both nuclear-cytoplasmic mRNA export and translation initiation (Figure 4, Table 1) [58,73].

**Table 1:** Table reporting eIF4E mutants often used to study eIF4E function.

	eIF4E mutant	eIF4E activity		Effect
		Cap- dependent Translation	Nuclear export	
Binding to the CAP	W56A	Inhibited	Inhibited	Prevents m7G Cap-binding [58,64,73]
Binding to EFBPs	W73F	Inhibited	Not altered	Prevents binding to eIF4G [68,73]
	Y76A	-	-	Reduce binding to Z-protein [74]
	W130F	-	-	Altering Cap-site conformation [74]
	S53A	Not altered	Impaired	Not well characterised [61–63]
	L134R	-	-	Affects protein conformation [8]
	W73A	Inhibited	Not altered	Prevents binding to eIF4G [60]
	V69/W73A	-	Impaired	Reduced association with LRPPRC [60]
Post- Translational Modifications	S209 / T210A^1^	Not altered	Reduced up 50% (*in vivo*)	Impairs eIF4E phosphorylation by MNK1/2 [7,77,78]

Mutations are grouped considering their impact to eIF4E function. For each mutation, effects on eIF4E conformation, cap binding, PPIs and post-translational modifications, are reported. L134R affects site 2 and protein conformation, L85R affects conformation of the flexible loop inactivated by compound 4 binding.

1 S209 is the site of MNK phosphorylation but mutation to S209A results in phosphorylation of neighbouring T210 [75,76]. Therefore, to accurately evaluate the role of S209 phosphorylation, a double S209A/T210A mutation was engineered [77]. Created in BioRender. https://BioRender.com/m21j443.

eIF4G interaction with the dorsal surface of eIF4E can further increase eIF4E cap-affinity by approx. two fold, which is counteracted through competitive binding of 4E-BP to an overlapping binding site (reviewed in [7]). Residues V69, W73, L131 and G139 on the dorsal surface of eIF4E are critical for interactions with eIF4G or 4E-BPs [68,79,80]. Substituting tryptophan at position 73 with phenylalanine (W73F) yields an eIF4E mutant unable to integrate into the eIF4F complex, thereby inhibiting cap-dependent translation without affecting mRNA transport [73] (Figure 4 , Table 1). In mouse lymphoma models, wildtype (WT) eIF4E accelerated tumour development, while tumours expressing W56A or W73F mutants grew more slowly, highlighting that the oncogenic role of eIF4E requires cap-binding and translation initiation through binding to eIF4G [73].

The precise molecular mechanism by which eIF4F promotes recruitment of the ribosomal pre-initiation complex to mRNA prior to 5' UTR scanning remains unclear. Two models are proposed: 1) a slotting model, where the mRNA directly inserts into the ribosome’s mRNA-binding cleft with minimal structural remodelling, or 2) a threading model, where mRNA is actively fed through a narrow channel in the 40S ribosome, potentially utilising the eIF4A1 RNA helicase and requiring conformational changes (reviewed in [81]). A related question is whether the 5' mRNA cap and eIF4E remain tethered to the small ribosomal complex during 5’ UTR scanning. The threading model requires the release of eIF4E from the cap and allows multiple ribosomal complexes to scan the same 5’ UTR. In contrast, a tethered eIF4E would restrict scanning to a single ribosomal complex per mRNA 5' UTR. Recent cryo-EM studies of the 48S ribosomal complex did not detect eIF4E in post-cap recognition complexes, suggesting eIF4E releases the mRNA cap before or upon start-codon selection [81–83]. However, further research is needed to pinpoint the precise timing and the mechanistic relevance of eIF4E release to mRNA binding and scanning.

eIF4E activity can be regulated through secondary interactions on its dorsal surface. Proteins with the Really Interesting New Gene (RING) domain, such as PML and the arenaviral Z-protein, induce conformational changes in eIF4E that reduce cap-binding affinity by ~100 fold [74]. In contrast, interactions with eIF4G or 4E-BP1 enhance the cap-binding affinity of eIF4E [79]. While the Z-protein and eIF4G have binding sites that partially overlap, there are also additional distinct regions of interaction [74]. Mutations that reduce Z-protein binding (Y76A or W130F) do not affect eIF4G interactions, demonstrating that different proteins can have distinct eIF4E-binding modes with different regions on the dorsal surface [74](Figure 4, Table 1). Further studies on the Z-eIF4E interaction could provide valuable insights into viral infection mechanisms [84]. Moreover, the Z-protein binding region of eIF4E may represent an additional druggable site on the eIF4E structure [63].

Post-translational modifications (PTMs) also tightly regulate eIF4E activity. Phosphorylation of eIF4E at S209, mediated by MNK kinases, has been shown to enhance eIF4E activity in response to stimuli such as stress and mitogens [77,85,86]. The transformation and tumourigenesis abilities of elevated phospho-eIF4E are completely abrogated upon S209 mutations [77]. Furthermore, S209 mutations reduce eIF4E-mediated mRNA export by up to 50%, possibly due to loss of association with 4ESE-binding proteins resulting in impaired transport of oncogenic mRNA transcripts and activation of stress responses [7,77]. However, although S209 phosphorylation is increased in various tumours, its functional significance remains unclear, as some studies suggest it is not essential under normal physiological conditions [78,87,88]. These observations are further complicated by potentially compensatory phosphorylation of a neighbouring T210 in S209A mutants of eIF4E [75,77].

In addition to phosphorylation, sumoylation of eIF4E enhances translational activity by promoting dissociation from 4E-BP1, thus facilitating eIF4F complex formation [67,89]. Sumoylation which follows S209 phosphorylation induces a secondary conformational change in eIF4E, increasing affinity for eIF4G [90,91]. These findings underscore the complexity of eIF4E regulation by PTMs and suggest that selectively targeting these pathways may offer novel therapeutic strategies [89–91].

Cap-dependent translation initiation can also proceed through eIF4E-independent mechanisms. One such pathway involves the cap-binding complex (CBC), which binds the mRNA cap and recruits CTIF as a scaffold protein, analogous to eIF4G recruitment in eIF4E-dependent translation (Figure 3B) [92]. Both eIF4G and CTIF share a conserved MIF4G domain (middle domain of eIF4G), which serves as a bridging module for eIF3 complex recruitment [92–94]. Several studies suggest that CBC plays a critical role in initiating the first ‘pioneer’ round of protein synthesis [92].

Additionally, a subset of mRNAs bypasses eIF4E and employs an alternative eIF3D-dependent mechanism to initiate translation, particularly under stress conditions or mTOR inhibition, when global translation is suppressed [93]. Under normal circumstances, eIF3D functions within the eIF3 complex to facilitate 40S ribosome recruitment. However, in its non-canonical role, eIF3D can directly bind 5' UTRs or the mRNA cap using a unique cap-binding domain structurally distinct from that of eIF4E [93]. Although it remains unclear whether this non-canonical activity still requires the eIF3 complex, it does involve DAP5 (eIF4G2), a homolog of eIF4G1 that lacks the eIF4E-binding domain and thus cannot mediate canonical cap-dependent translation (Figure 3C) [94]. This alternative pathway allows for selective translation of specific mRNAs during translational repression, contributing to processes such as oncogenesis, immune responses and viral replication [93,94].

### Targeting eIF4E in cancer

Components of the eIF4F complex, particularly eIF4E, are strongly implicated in cancer through gene amplification, oncogene regulation and direct involvement in cancer progression [95–103]. Deregulated or elevated expression of eIF4E is associated with a broad spectrum of human cancers including skin [104], breast [105], lung [106], gastric [107], colon [108], prostate [109] and the haematopoietic system [67,99]. High eIF4E levels alongside 4E-BP1 hyperphosphorylation, which enhances eIF4E availability for eIF4F complex formation, are associated with decreased overall survival in both breast and prostate cancers [105,109].

As described in the previous section, there are other examples of initiation factors, including eIF3D or NCBP2 – which forms a cap binding complex (CBC) with its adaptor NCBP1 – that can initiate cap-dependent translation independently of eIF4E (Figure 3C) [93,110]. However, targeting eIF4E still holds significant therapeutic promise, since beyond its established role in initiating protein synthesis, the oncogenic activity of eIF4E may also extend to the nuclear export of specific mRNAs possessing oncogenic properties as well as serving as a key convergence point for three major oncogenic pathways: c-MYC, PI3K/mTOR and RAS/RAF/MEK signalling via MNK kinases [99,111,112]. A major node of these oncogenic pathways is c-MYC which controls transcription of many genes, including eIF4E, required to induce malignant transformation [113]. In turn, eIF4E regulates translation of c-MYC, ultimately creating a feedforward loop that, when dysregulated, promotes tumourigenesis [103,114]. eIF4E overexpression and hyperactivation is sufficient to induce tumourigenic transformation of fibroblasts [115] and primary epithelial cells [116] both *in vitro* and *in vivo*. Furthermore, transgenic mice overexpressing eIF4E develop cancers of distinct histological origin including lymphomas, hepatomas, lung adenocarcinomas and hepatocellular adenomas, many of which are among the human cancers associated with eIF4E overexpression [99,117].

A critical question is whether therapeutic strategies targeting eIF4E can produce consistent effects in cancer cells while maintaining a therapeutic window that spares normal tissues. Exploring diverse approaches targeting eIF4E is therefore essential. The dependency of cancer cells on eIF4E, coupled with the presence of other cap-binding proteins which could compensate for loss or inhibition of eIF4E activity, suggests the possibility of a therapeutic window. A haplo-insufficient mouse model has demonstrated that partial loss of eIF4E can indeed be tolerated [118]. In these mice, loss of one eIF4E allele and the resulting reduction in eIF4E protein expression did not impair embryonic development or maintenance of global protein synthesis levels. Despite the tolerance to reduced eIF4E expression, these mice exhibited strong resistance to cellular transformation and tumourigenicity induced by oncogenes such as H-RAS, further supporting the existence of a therapeutic window [118]. Table 2 summarises current efforts to inhibit eIF4E using direct and indirect methods, including small-molecule or peptide mimetics, disruption of PPIs and improved antisense delivery [11].

**Table 2:** Overview of different approaches to inhibit eIF4E and their current development stage (D & D, discovery and development). Clinical trial status at time of reporting March 2025, (https://clinicaltrials.gov).

Primary mechanism of action	Name	Type of agent	Development stage	Reference
**4E-BP1 phosphorylation**				
**mTORC1-specific**	Rapamycin	Small molecule	Phase II	[119,120]
	Everolimus	Small molecule	Approved	[121]
	Temsirolimus	Small molecule	Approved	[122]
	Ridaforolimus	Small molecule	Phase III	[123]
**mTORC-kinases**	Torin 1	Small molecule	D & D	[124]
	Torin 2	Small molecule	D & D	[125]
	PP242	Small molecule	D & D	[126]
	PP30	Small molecule	D & D	[126,127]
	MLN0128	Small molecule	Phase II	[128]
	AZD8055	Small molecule	Completed	[129]
	OSI-027	Small molecule	Completed	[130]
	Rapalink-1	Small molecule	D & D	[131]
**PI3K signalling pathway**	PI-103	Small molecule	D & D	[132]
	Alpelisib	Small molecule	Phase IV	[133]
**AKT**	MK-2206	Small molecule	Phase II	[134]
	Ipatasertib	Small molecule	Phase III	[135]
	Capivasertib	Small molecule	Approved	[136]
**CDK4**	Palbociclib	Small molecule	Approved	[137,138]
**eIF4E-dependent**				
**RNA export - XPO1**	Selinexor	Small molecule	Approved	[139,140]
**eIF4E phosphorylation - MNK1**	Tomivorsetib	Small molecule	Phase II	[141,142]
	Cercosporamide	Small molecule	D & D	[143]
	CGP57380	Small molecule	D & D	[144]
	Bay1143269	Small molecule	Terminated	[145,146]
	VNLG-152R	Small molecule	D & D	[147]
**eIF4E:eIF4G interaction**	4EGI-1	Small molecule	D & D	[148]
	4E1RCat	Small molecule	D & D	[149]
	Compound **4**	Small molecule	D & D	[8]
	i4EG-BiP	Small molecule	D & D	[150]
	d4E-6	PROTAC	D & D	[150]
	d4E-2	PROTAC	D & D	[150]
	HCS-4E-BP1	Peptidomimetic	D & D	[151]
	mHCS-4E-BP1	Peptidomimetic	D & D	[152]
	LacB-4E-BP1	Peptidomimetic	D & D	[153]
**mRNA cap-binding**	Compound **33**	Small molecule	D & D	[154]
	4EI-1	Small molecule	D & D	[155]
	Ribavirin	Nucleoside analogue	Phase II	[156]
	Bn7gdp-based PROTAC	PROTAC	D & D	[157]
	Cap analogue 5	Prodrug	D & D	[158]
	Cap-binding covalent inhibitor 12	Small molecule	D & D	[159]
**eIF4 expression**	LY2275796	Oligonucleotide	Completed	[160]
	ISIS 183750	Oligonucleotide	Completed	[161]

Antisense oligonucleotides targeting eIF4E have demonstrated the ability to inhibit human breast cancer xenograft growth [162]. In clinical studies, ISIS 183750, an eIF4E antisense, successfully reduced eIF4E expression. However, Phase II clinical trials did not show objective tumour responses, and further development was halted [160,161]. The clinical application of antisense technology has been limited by inefficient delivery. However, advancements in aptamer conjugates, nanoparticles, or lipid-based delivery may overcome these challenges, potentially reviving interest in antisense strategies.

Alternatively, targeting eIF4E indirectly, either through inhibition of the eIF4F-associated RNA helicase eIF4A or upstream kinases has demonstrated altered translation of oncogenic mRNAs, suggesting a tailored therapeutic strategy [99]. One approach is to target the mRNA export activity of eIF4E through its dependence on the nuclear export protein XPO1. Selinexor is a selective small molecular inhibitor of CRM1/XPO1 transport that has been demonstrated in pre-clinical and clinical studies to impair eIF4E protein export and eIF4E-dependent mRNA export and is FDA approved for use in relapsed or refractory diffuse large B-cell lymphoma or in combination with dexamethasone or bortezomib for the treatment of relapsed or refractory multiple myeloma [57,139,140,163,164].

One class of eIF4E modulators that have advanced to the clinic are MNK inhibitors, which block the phosphorylation of multiple downstream factors, including hnRNPA1, PSF, cPLA_2_ and Spry2 and eIF4E [165]. Tomivosertib (eFT-508) is a potent, highly selective and orally bioavailable inhibitor of MNK1 and MNK2. In Phase I/II clinical trials, Tomivosertib only showed modest efficacy as a single agent, and further clinical development as a single agent was halted. Preclinical studies suggested synergistic effects with immune checkpoint inhibitors; however, clinical trials with combinations found limited improvement in overall response rates compared with checkpoint inhibitor monotherapy [166]. Nonetheless, several genetically engineered mouse model studies have suggested alternative combination strategies with Tomivosertib. For example, Knight and colleagues demonstrated that while inhibiting S209 phosphorylation alone did not affect K-RAS-driven tumour growth, it significantly sensitised tumours to the mTORC1 inhibitor, rapamycin [167]. Furthermore, Tomivosertib showed strong anti-tumour effects in pancreatic cancer mouse models by impairing ketogenesis during dietary fasting, highlighting its potential in specific metabolic contexts and revealing a new therapeutic strategy to treat pancreatic cancer [168].

An alternative strategy involves targeting either the mRNA cap-binding process or PPIs with critical partners like eIF4G. However, both approaches present unique challenges, such as the polar nature of the cap-binding sites or the extensive binding interface between eIF4E and eIF4G. Ribavirin, a guanosine analogue originally developed as an antiviral agent, has also shown activity in early clinical trials as a monotherapy and in combination regimens [7,169–172]. Preclinical and clinical data have indicated that Ribavirin competes with eIF4E cap-binding, stimulating re-localisation from a predominantly nuclear location to the cytoplasm, with relapse linked to continued nuclear localisation or re-localisation from the cytoplasm [170]. While this evidence supports eIF4E modulation as a key factor in the mechanism of action of Ribavirin, it is important to note that this drug interacts with multiple molecular pathways, all of which could contribute to its cancer-specific mechanism of action [11,169,173].

Preclinical and clinical studies with drugs such as Ribavirin, Selinexor and MNK1/2 or mTORC inhibitors have demonstrated that inhibiting eIF4E activity is tractable. However, their use as chemical tools to complement genetic knockout strategies is limited, as these agents either exhibit poly-pharmacology targeting multiple proteins including eIF4E - or act on distal regulators of eIF4E but have additional downstream targets. Thus, it remains challenging to unequivocally attribute any of their anticancer effects to eIF4E inhibition alone. A more effective approach for probing eIF4E function and developing therapeutics is to directly and selectively target eIF4E itself. Over the past two decades, biochemical screening of compound libraries has identified proof-of-concept pharmacophores [174–177]. Lead compounds with low-nanomolar biochemical potency can block mRNA cap binding, while others inhibit the eIF4E:eIF4G interaction. However, these tool compounds lack key features of high-quality chemical probes, such as confirmed selectivity, cellular potency and structurally related inactive controls, in addition to favourable physiochemical properties like solubility [174,178]. To date, no reports indicate that these early tool compounds have progressed into drug-like chemical series with improved pharmaceutical properties [178].

We recently addressed these challenges by screening a small fragment library using a combination of ligand-observed NMR and X-ray crystallography to identify potentially druggable sites [8]. This unbiased approach differed from previous screening methods that specifically targeted the cap-binding or the interaction between eIF4E and eIF4G peptide sequences [154,175–177]. Fragment hits were identified that bound to the mRNA cap-binding site and a second site (site **2;** Figure 4) on the lateral surface of eIF4E proximal to both the canonical and non-canonical eIF4G/4E-BP1 interaction sites described by both Sekiyama and Gruner [8,70,179]. Through iterative rounds of structure-based design, the site **2** binding fragment hits were optimised to compound **4**, that binds eIF4E with a 90 nM *K*_*d*_. Binding of compound **4** induces a conformational change to the α1-helix and flexible loop proximal to the non-canonical eIF4G/4E-BP1 binding site that could potentially affect these eIF4E interactors (Figure 4). Consistent with this idea, compound **4** disrupted the eIF4E:eIF4G interaction and inhibited translation in cell lysates.

At the time of screening, site **2** had unknown functional relevance, although more recently Fischer and colleagues reported a biphenyl-derivative of 4EGI-1 (i4EG-BiP), which binds in the same region but makes different interactions to those identified for compound **4** [70,148,150]. Compound **4** could displace binding of full-length eIF4G in cell lysates (EC_50_ ≈ 1.5 μM) similar to 4EGI-1 and i4EG-BiP (56 and 67 μM IC_50_ respectively in an eIF4E:eIF4G peptide fluorescence polarisation assay). Interestingly, displacement of eIF4G from eIF4E by compound **4** did not result in the recruitment of 4E-BP1 to eIF4G that was reported following 4EGI-1 or i4EG-BiP treatment [148]. This suggested compound **4** has a different binding mode to 4EGI-1 and i4EG-BiP and warrant further in-depth studies to understand the complexities of 4E-BP1 or eIF4G binding to eIF4E and the impact of small molecules.

Compound **4** could bind to eIF4E in intact cells at 1–2 μM; however, the activity of compound **4** dropped significantly in live-cell assays of protein synthesis. Cell-based mutational analysis showed that simultaneous disruption of both the canonical and non-canonical eIF4G binding sites was necessary to elicit a strong cellular response from mutation or compound [8]. These findings underscore the importance of structural insights and ligand-binding site mutations to guide the understanding of eIF4E function and the development of potent small-molecule inhibitors or degraders targeting eIF4E.

### eIF4E in human disease: roles beyond cancer

While eIF4E is primarily studied for its role in cancer, its deregulation and functional involvement also extends to several non-cancerous human diseases such as autism, Alzheimer’s disease and type 2 diabetes [76,180–193]. These conditions highlight the broader implications of eIF4E in regulating translation and RNA metabolism, underscoring its relevance in various pathological contexts and its broad physiological importance beyond oncogenesis. By regulating cap-dependent translation, eIF4E affects processes critical for neuronal health, metabolism, cardiovascular function, immune responses and development. These insights also position eIF4E as a potential therapeutic target in diverse pathological contexts, including neurodegenerative, metabolic and inflammatory diseases (Table 3).

**Table 3:** Summary of the potential influence of eIF4E in non-cancerous human disease.figure

Disease type	Role of eIF4E	Pre-Clinical targeting	Clinical trials
**Neurodegenerative diseases**			
Fragile X syndrome	Abnormal signalling of the mTOR pathway regulates eIF4E activity, enhanced translation is linked to synaptic dysfunction and cognitive impairment [180]	Rapamycin, Tomivosertib, Cercosporamide, Metformin [194–197]	Metformin [198]
Autism spectrum disorders	Enhanced signalling regulating eIF4E/eIF4G interaction affects synaptic protein synthesis [182]	4EG-1 [181,182]	
Alzheimer’s Disease	Deregulated synthesis of synaptic proteins and accumulation of misfolded proteins, exacerbating neurodegeneration [183]	CGP 57380 [183]	
**Metabolic disorders**			
Type 2 diabetes mellitus	Enhanced translation of transcripts involved in inflammation and lipid metabolism, contributing to insulin resistance [184,185]	Metformin, Rapamycin [199–201]	Metformin [202–204]
Obesity	Increased adipogenesis and inflammatory cytokine production, linked to obesity-related metabolic dysfunction [186]	Tomivosertib, COH-SR4, Metformin [186,205,206]	Metformin [207]
**Cardiovascular diseases**			
Cardiac hypertrophy	Abnormal protein synthesis in cardiomyocytes, driving pathological hypertrophy and heart failure [187]	Lithium, Rapamycin [208–211]	
Atherosclerosis	Regulates the translation of pro-inflammatory mRNAs encoding cytokines and adhesion molecules that contribute to vascular inflammation and plaque formation [188]	Rapamycin [212]	
**Infectious diseases**			
Viral replication	Some viruses e.g. HIV, influenza and hepatitis C virus hijack the host translation machinery and exploit eIF4E to enhance their replication [189]	Ribavirin [213,214]	Ribavirin [215]
Immune response	eIF4E regulates the translation of immune-associated transcripts, affecting the production of cytokines and interferons during infection and impacting on immune defences [190]	Ribavirin, Rapamycin [216–219]	Rapamycin [220,221]
**Inflammatory diseases**			
Chronic inflammatory disorders	eIF4E regulates the translation of pro-inflammatory cytokines and chemokines with roles in conditions such as rheumatoid arthritis and inflammatory bowel disease [192]	Rapamycin [222–224]	
**Developmental and genetic disorders**			
Vanishing white matter disease	Leukodystrophy that involves mutations in translation initiation factors, includes eIF4E interactors, disrupting protein synthesis critical for myelin production [76,191]	Transcranial magnetic stimulation [225]	
Intellectual disability and microcephaly	Rare mutations in eIF4E and associated factors disrupt cap-dependent translation, impairing brain development and function [193]		

## Summary

Most research has focused on eIF4E’s canonical role in translation initiation. However, given more recent insights into its broader functions, it is essential to revisit these studies with an expanded perspective. For example, a critical factor may be the balance between the cytoplasmic and nuclear roles of eIF4E in cancer cells and other disease models. Cellular transformation often leads to elevated transcription rates, which, in turn, may increase the demand for mRNA nuclear export. This heightened burden on mRNA export could reduce the availability of eIF4E for protein synthesis initiation, as it becomes sequestered for export functions. This phenomenon may also align with observations over the past decade that cancer cells frequently utilise eIF4E-independent mechanisms to sustain protein synthesis, employing both cap-dependent and cap-independent pathways [226]. Increased use of eIF4E-independent pathways in cancer cells may also reflect the low levels of altered protein expression detected in global proteome profiles of cancer cells following loss of eIF4E by siRNA or eIF4E-degron approaches [8,227]. Further investigations are needed to better distinguish the contributions of eIF4E’s multiple roles in disease pathology. The availability of potent, selective tool compounds to complement existing genetic approaches will greatly facilitate these studies. Moreover, a comprehensive understanding of the pathways involving eIF4E is fundamental to identifying the best therapeutic approaches for effectively targeting this critical protein.

**Perspectives**

- Eukaryotic translation initiation factor 4E (eIF4E) is a key component of the eIF4F translation initiation complex and a regulator of nuclear export and mRNA processing. This multifunctional role is crucial for protein expression, cell survival and proliferation and is linked to human disease, particularly cancer, making eIF4E a promising therapeutic target.
- A deeper understanding of eIF4E’s structure, function and binding partners will help to identify new strategies for its direct modulation. This will include exploiting synthetic lethal interactions with eIF4E inhibition or designing drug combinations to enhance treatment efficacy.
- Identifying potent and selective chemical probes targeting eIF4E will provide valuable tools to complement existing biophysical, biochemical and genetic approaches to study eIF4E in normal and disease models. The availability of high-quality chemical tools will also provide starting points for the development of novel eIF4E-targeted therapeutics.

## Open Access

This article has been published open access under our Subscribe to Open programme, made possible through the support of our subscribing institutions, learn more here: https://portlandpress.com/pages/open_access_options_and_prices#conditional

## Abbreviations

APA: alternative polyadenylation
CPA: cleavage and polyadenylation
PAS: polyadenylation signal
PPI: protein-–protein interaction
eIf4E: eukaryotic translation initiation factor 4E
m^7^G-cap: methyl-7-guanosine cap
